# Expected increase in staple crop imports in water-scarce countries in 2050

**DOI:** 10.1016/j.wroa.2018.09.001

**Published:** 2018-10-05

**Authors:** Hatem Chouchane, Maarten S. Krol, Arjen Y. Hoekstra

**Affiliations:** a Water Engineering & Management, University of Twente, Enschede, the Netherlands; b Institute of Water Policy, Lee Kuan Yew School of Public Policy, National University of Singapore, 259770, Singapore

**Keywords:** Global trade, Food security, Staple crops, Water-scarcity, Population growth

## Abstract

Population growth paired with growing freshwater scarcity in various parts of the world will reduce the potential of food self-sufficiency in many countries. Today, two thirds of the global population are already living in areas facing severe water scarcity at least one month of the year. This raises the importance of addressing the relationship between water availability and food import in water-scarce countries. Net import of staple crops (including cereals, roots, and tubers) is analysed in relation to water availability per capita for the period 1961–2010, considering five decadal averages. The relation found is used, together with the population growth scenarios from the United Nations, to project staple crop imports in water-scarce countries for the year 2050. As a result of population growth in water-scarce countries alone, global international trade in staple crops is projected to increase by a factor of 1.4–1.8 towards 2050 (compared to the average in 2001–2010), in order to meet the staple food needs of the 42 most water-scarce countries in the world.

## Highlights

• Net import of staple crops is analysed in relation to water availability per capita.
• Future international staple crop trade is projected using population growth scenarios.
• Global trade in staple crops will increase by a factor of 1.4–1.8 towards 2050.

## Graphical abstract

**Figure:**
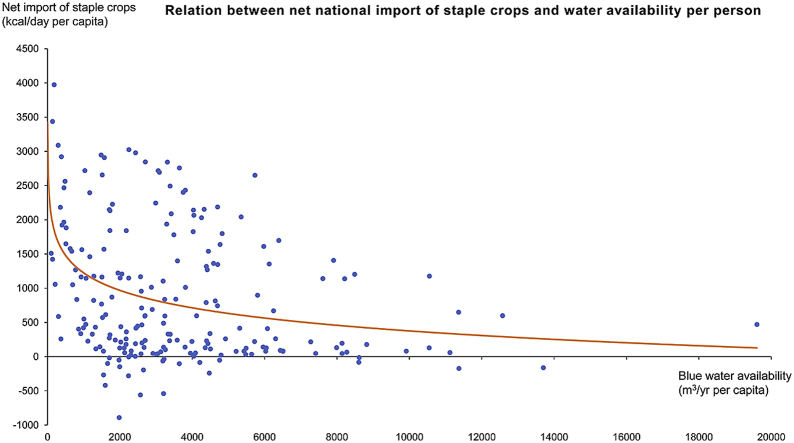

## 1 Introduction

Water scarcity is a major challenge in the coming decades, particularly for food production (Davis et al., 2017). An estimated 4.0 billion people are living under conditions of severe water scarcity for at least 1 month of the year, 3.3 billion for at least 3 months, and 1.8 billion at least half a year (Mekonnen and Hoekstra, 2016). The increasing population and the changing pattern of water availability that results from global warming reduce the potential of sufficient food production in many countries (Godfray et al., 2010). Given that already today most water-scarce countries rely on food imports, the question is how much the water-scarcity driven global demand for food imports may grow.

According to the medium projection of the United Nations, the world population will reach 9.7 billion by 2050 and exceed 11 billion by 2100 (United Nations, 2015). Africa and Asia will have the highest population growth between 2015 and 2050, with projected population increases of 52% and 17%, respectively (United Nations, 2015). These two continents already have the highest undernourishment prevalence levels, viz. 20% and 12% of their total population, respectively (FAO et al., 2015). Population growth and climate change are major variables affecting future water demand and scarcity (Vörösmarty et al., 2000) and thus food security (Hanjra and Qureshi, 2010). While the green revolution helped in the past to feed a growing global population, there is a growing concern about the future (Gilland, 2002). Climate change is expected to aggravate the situation and threatening food security by altering spatial and temporal rainfall patterns, reducing crop yields in various vulnerable regions (Parry et al., 2004) and lowering food security particularly in sub-Saharan Africa and South Asia (Schmidhuber and Tubiello, 2007). However, it has been widely acknowledged that when it comes to water scarcity, population growth is a bigger driver than climate change (Arnell, 2004; Gerten et al., 2011; Schewe et al., 2014; Vörösmarty et al., 2000).

Food insecurity in water-scarce countries can partially be mitigated through improving water productivity in crop production (Kate et al., 2013), through better irrigation and agricultural management practices (Chukalla et al., 2015; McLaughlin and Kinzelbach, 2015; Tilman et al., 2011). Food import may be another solution to fill the gap between demand and supply from domestic food production in many countries and especially in water-scarce countries. Such imports go along with virtual water import (Allan, 1998), externalising water consumption. Virtual water trade could be a solution to cope with physical water scarcity if water-scare countries import their water-intensive food needs from water-abundant countries (Aldaya et al., 2010; Chapagain and Hoekstra, 2008; Hoekstra and Hung, 2005).

During the past decade, a growing number of virtual water trade studies has become available (Antonelli and Sartori, 2015; Hoekstra, 2017), some focussed on quantification and others on analysing policy implications, but surprisingly little effort has been made to correlate virtual water import to water scarcity. Most notably, for countries in Asia and Africa, Yang et al. (2003) investigated the relationship between cereal import and per capita water resources availability for a period of ten years. A water scarcity threshold of about 1500 m^3^/y per capita was identified, below which cereal import of a country increases exponentially with the decline of per capita water availability. Above it, no direct relationship was found between cereal import and water endowment. A weakness of this study was that staple crops other than cereals, like starchy roots and tubers (e.g. cassava, potatoes, sweet potatoes and yam) were not included, while many developing countries depend on these other staple crops. Besides, it is more meaningful to consider staple food import in terms of kcal per capita than in terms of kg per capita. No follow-up has been given after this valuable initial study from Yang et al. (2003), while good insight into water-scarcity driven demand for food import could be useful to project future needs of the many countries that face water scarcity already today.

This paper aims to study the relation between import of staple foods (including cereals, roots and tubers) and water scarcity with a long-term and global perspective. The net import of staple crops in kcal/y per capita is analysed in relation to water availability per capita for the period 1961–2010, considering five decadal averages. The relation found is used together with the low, medium and high population growth scenarios from the UN (United Nations, 2015) to project future staple crops import in water-scarce countries for the year 2050. Additionally, we investigate the uncertainties related to the three population scenarios and related to the regression analysis.

## 2 Material and methods

Countries have been selected based on three criteria. The selection contains countries with a maximum average blue water availability of 5000 m^3^/y per capita in the period 2001–2010. Excluded are countries for which more than 50% of total domestic supply of staple crops is for feed, seed, processing or other uses and not for food supply. Excluded are also countries with a population smaller than 500.000 inhabitants in the year 2010. This resulted in a selection of 42 countries.

In total, 13 staple crops were selected: barley, cassava, maize, millet and products, oats, potatoes, rice, rye, sorghum, soybeans, sweet potatoes, wheat and yams. The selection is based on the main produced, consumed and traded crops globally. The selected crops account to 45% of total crop quantity produced, 50% of total food supply in kcal/day per capita, 44% of total crop import and 43% of the total crop export quantities in 2010 (FAO, 2015).

Gross imports and exports of staple crops in tonne/y per country for the period of study (1961–2010) were taken from the FAOSTAT database (FAO, 2015). Net imports of staple crops in kg were converted into kcal using conversion rates (Supplemental Table 1). Per country, average net import of staple crops per decade was calculated, for each of the five decades in the study period and net import per capita was calculated using decadal average population data from the UN (United Nations, 2015).

The total blue water availability per capita per country was derived from the FAOSTAT database (FAO, 2015). Water availability is taken as the total renewable water resources (TRWR), which is the sum of internal and external renewable water resources of a country (FAO, 2003). We added, per country, the yearly produced desalinated water. Although desalinated water is mostly used in other sectors than agriculture, it helps in reducing the overall pressure on freshwater resources. In this way, countries that use desalinated water may dedicate a larger share of their fresh water to agriculture. The TRWR values, representing 30-year averages (1961–1990), are assumed to be constant per country over the period of study and divided by decadal averages of population to obtain average water availability per capita per decade.

Regression modelling is used to analyse the relation between the blue water availability per capita (y) and the net import of staple crops per capita (x). Statistical model testing proved a logarithmic relation to yield the best fit amongst standard functional options for the relation. The model was extended to the equation $\;y=\text{a}\;\text{log}x+b+\overset{42}{\underset{i=2}{\sum }}c_{i}\cdot d_{i}$ including country-specific biases *c*_*i*_, implemented by including country-specific dummy variables *d*_*i*_. Statistically, country-specific biases were significant for most countries and the model extension improved the fitness of the model (higher R^2^).

The medium population growth scenario of the UN (United Nations, 2015) is used to obtain projections for the decrease in blue water availability per capita per country for the year 2050. The projected net import of staple crops per capita per country in 2050 (y) based on the blue water availability per capita per country for the year 2050 (x), is estimated using the equation $\;y=\text{a}\;\text{log}x+b+\overset{42}{\underset{i=2}{\sum }}c_{i}\cdot d_{i}+e_{i}$, whereby the values for a, b and c are taken according to the best-fitting curve for the last decade (2001–2010), and e_i_ is the difference between the curve and the value in the last decade for each country.

The impact of two types of projection uncertainties are estimated. The uncertainty in population growth is studied by considering UN's low and high population growth scenarios (United Nations, 2015). The uncertainty in the shape of the regression curve is estimated by varying the regression slope coefficient within its 95% reliability interval.

## 3 Results

### 3.1 Past blue water availability and net import of staple crops (1961–2010)

**Fig. 1:**
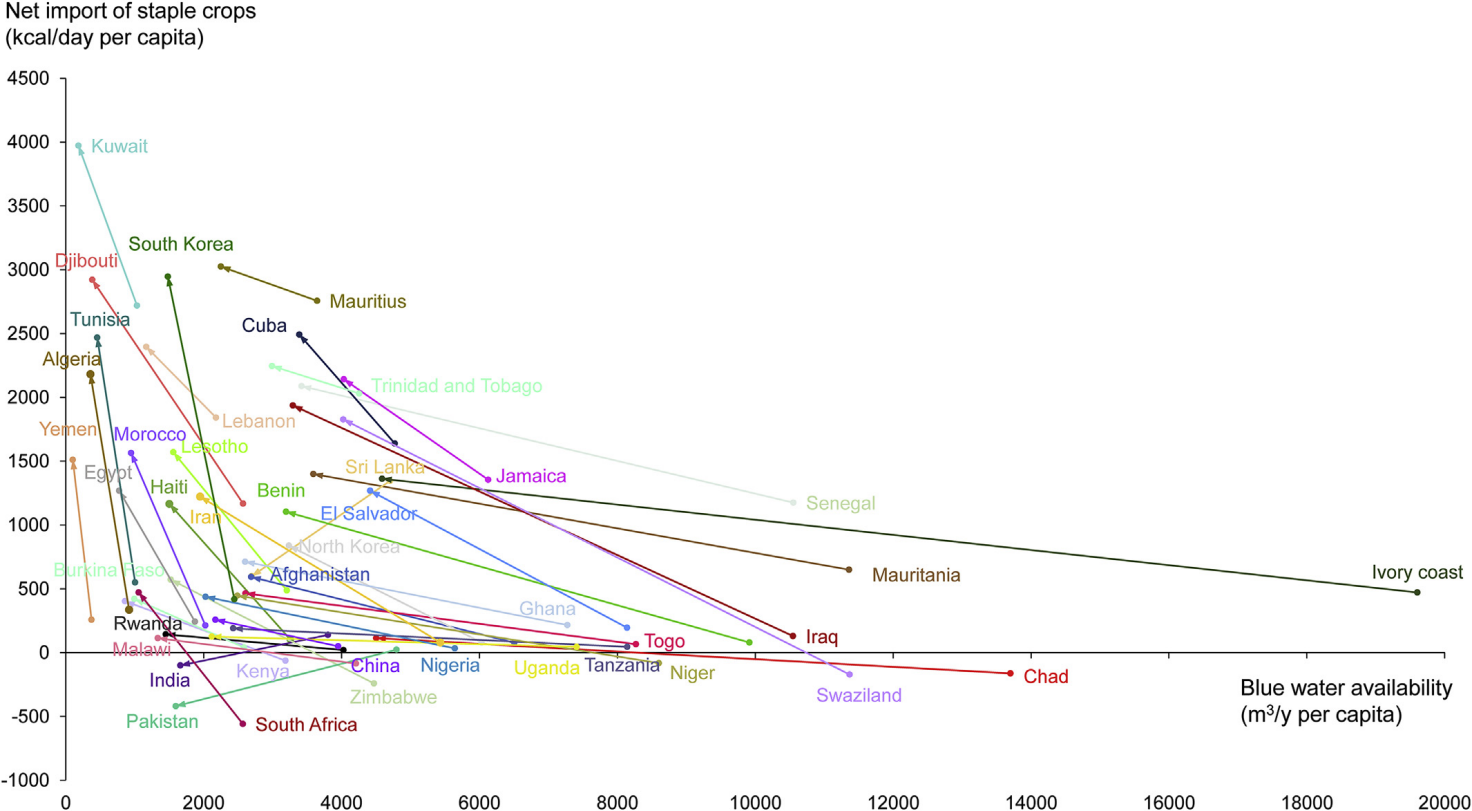
Change in the average blue water availability per capita and net import of staple crop per capita for the selected countries, from the period 1961–1970 to the period 2001–2010.

Changes in blue water availability and net import of staple crops from the period 1961–1970 to the period 2001–2010 for 42 selected countries show a clear general trend (Fig. 1). There is a continuous increase in net import of staple crops per capita with decreasing water availability per capita. The effect of decreasing water availability per capita on food import becomes more pronounced when water availability becomes less. The best fitting regression curve through all data points – when also including the data points for all five decades considered – follows a logarithmic shape as shown in the Supplementary Information (Supplemental Fig. 1).

India, Pakistan and Sri Lanka were the exceptions to the general pattern, with decreasing net staple food imports. While Sri Lanka remained a net importer of staple crops, India and Pakistan shifted to become net exporters. Some countries shifted from being net exporters of staple crops during the first decade to net importers in the last decade. This is the case for Chad, Malawi, Niger, Kenya, South Arica, Swaziland and Zimbabwe, where a growing domestic demand of staple crops due to population growth affected the countries’ capability to export and increased their import of staple crops.

**Fig. 2:**
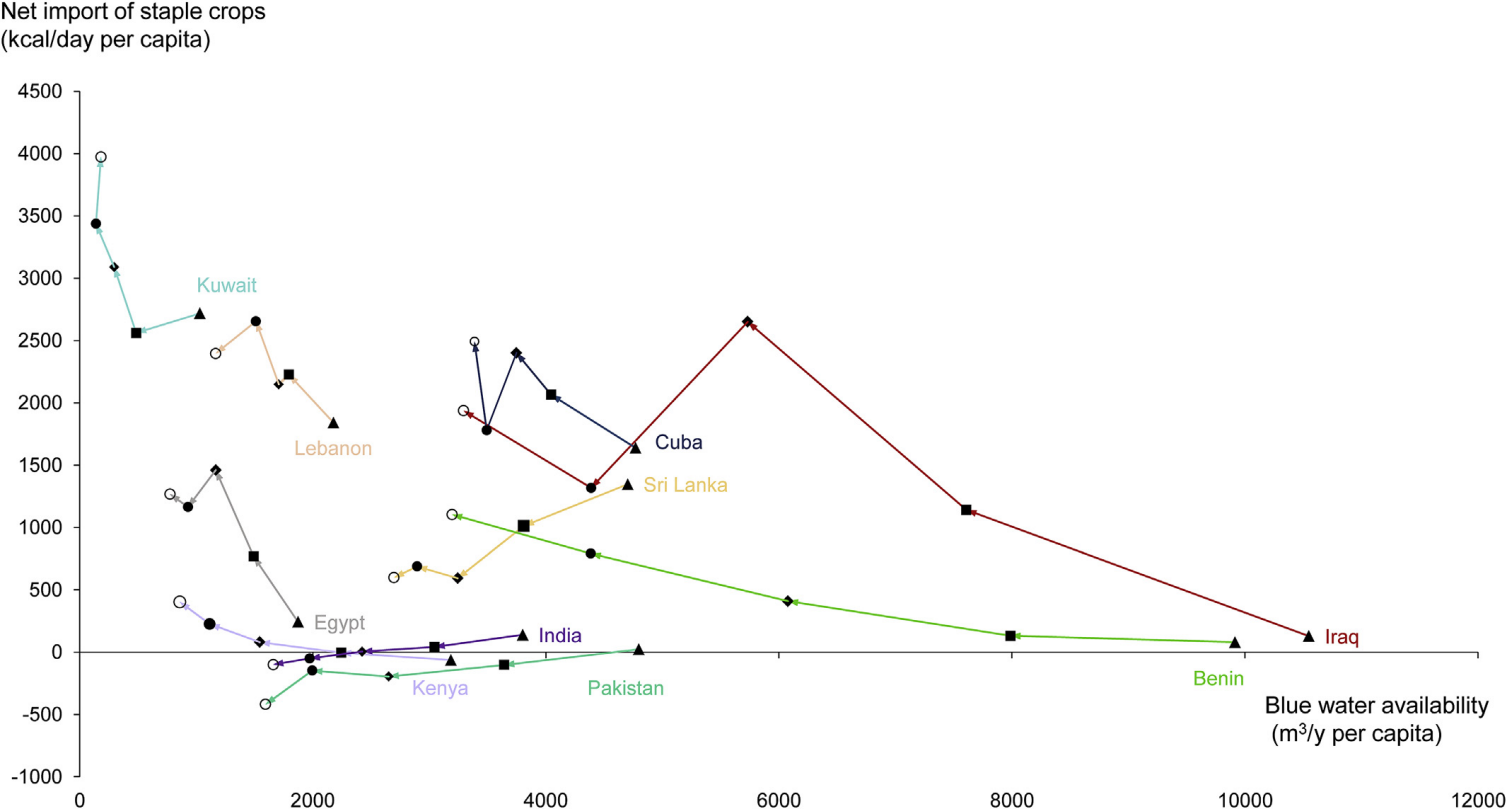
Trajectories of decadal average blue water availability per capita and net import of staple crop per capita over the 1960s, 1970s, 1980s, 1990s and 2000s for ten selected countries.

While Fig. 1 shows changes in blue water availability and staple crops import per capita between the initial and last period exclusively, hiding intermediate points in time, Fig. 2 shows all data for the five decadal averages for a few selected countries. This exemplifies that some countries shift gradually over time, while other countries show a bit more erratic behaviour. Each country has its specific underlying story.

Benin exhibits a progressive decrease in blue water availability and increase in the net import of staple crops consistent with the shape of the global regression curve. For Iraq, there was a drop in net staple food import between the 1980s and 1990s. The economic sanctions imposed on Iraq (1990–2003) after the Gulf war limited the imports of staple crops (Alnasrawi, 2001), which was partially compensated with increased national production but mostly resulted in reduced food supply. Food supply dropped by 32% between 1990 and 1991 and is still recovering two decades later (FAO, 2015). Kuwait has seen a large decline in its food supply as well, dropping by 25% between 1989 and 1990 and reaching its minimum in 1991 with 1900 kcal/day per capita (FAO, 2015). Opposite to Iraq, the food supply of Kuwait recovered already in 1995 (Supplemental Fig. 2). Therefore, Kuwait's food supply drop is not visible in the decadal averages appearing in Fig. 2, showing the country to follow the steep part of the regression curve at very low water availability. Cuba follows the trend of the global regression curve, but had a dip in net import of staple crops in the 1990s. The collapse of the Soviet Union resulted in deceasing Cuban imports due to its economic dependence (Perkins, 1993). Cuban imports of maize and wheat dropped by 85% and 36%, respectively, between 1990 and 1991 (FAO, 2015). Food supply decreased from 1989, reached its lowest level in 1993, and started to recover in 1996. In the 2000s, imports increased again. The data for Egypt show an anomaly for the 1990s as well, as shown by Yang et al. (2003). as well. The expansion in irrigated land of about 35% during that period increased the production of cereals, resulting in a decreased import of cereals per capita while total import continued to increase (Yang et al., 2003). The import dependency ratio of staple crops has decreased from 40% in average between the 1970s and 1980s to an average of 29% between the 1980s and 1990s (FAO, 2015). Yang et al. (2003) expected that the proportion of the imported cereals in the per capita consumption will again increase in the future as the country's water use is reaching its limit. This is confirmed in Fig. 2, which shows that one decade later, the net import of staple crops regained its increasing trend while the import dependency ratio of all staple crops has increased to 31% in average between the 1990s and 2000s. In the same way, Lebanon's import dependency ratio has dropped by 4% due to an increased production of barley, potatoes and wheat between the 1990s and 2000s, which explains the decrease in the net staple crops import for the last decade.

For the case of Kenya, a country that was net exporter of staple crops until 1977, the import dependency ratio has jumped from 3% during the 1960s to over 27% in the period 2001–2010 to keep pace with the growing domestic demand of staple crops. This is also partially explained by Kenya's policy to promote the production of cash crops (Gow and Parton, 1995) such as coffee and tea. While staple crop production has in average dropped by 25% between the 1960s and 1990s, coffee and tea production have grown by almost 1.5 and 5 times, respectively, in the same period (FAO, 2015).

India, Pakistan and Sri Lanka are the only three countries that do not follow the main trend line. Both India and Pakistan have even become a net exporter of staple crops despite their increasing water scarcity. In India, changes in trade mainly concern increases in rice and wheat exports. The green revolution has boosted the productivity in agriculture: average wheat yield increased from 0.8 tonne/ha in the 1960s to about 3 tonne/ha in the 1990s, and average rice yield doubled (FAO, 2015). India thus succeeded to become food self-sufficient, but at the expense of a rapid increase in the appropriation of water resources, leading to severe water depletion in many places (Mekonnen and Hoekstra, 2016). The intensive use of irrigation from groundwater and surface water has caused groundwater degradation in many districts of Haryana and Punjab, the largest contributing states to rice and wheat production in India (Singh, 2000). The irrigated area had been continuously increasing to maintain the food self-sufficiency policy. Similarly, Pakistan was one of the first beneficiaries of the green revolution in the 1960s, with intensification through the introduction of high-yielding varieties in wheat and rice, and the application of irrigation and fertilisers (Ali and Byerlee, 2002). This has led to negative environmental impacts such as salinization, overexploitation of groundwater, physical and chemical deterioration of the soil, and pest problems (Gupta and Seth, 2007). Sri Lanka, a country with decreasing imports during the five-decade period, is an example of a country with an agricultural policy aiming for food self-sufficiency in all crops and especially in the production of rice, the country's main staple crop. Due to a combination of high-yielding varieties, paddy expansion and increased use of irrigation and fertilizer, rice production in Sri Lanka has risen to meet almost 100% of its domestic demand (Davis et al., 2016). The production of rice has increased by a factor of almost five between 1961 and 2010 while the average yearly yield has increased from 1.9 tonne/ha to over 4 tonne/ha during the same period (FAO, 2015).

### 3.2 Projected blue water availability and net import of staple crops (2050)

**Fig. 3:**
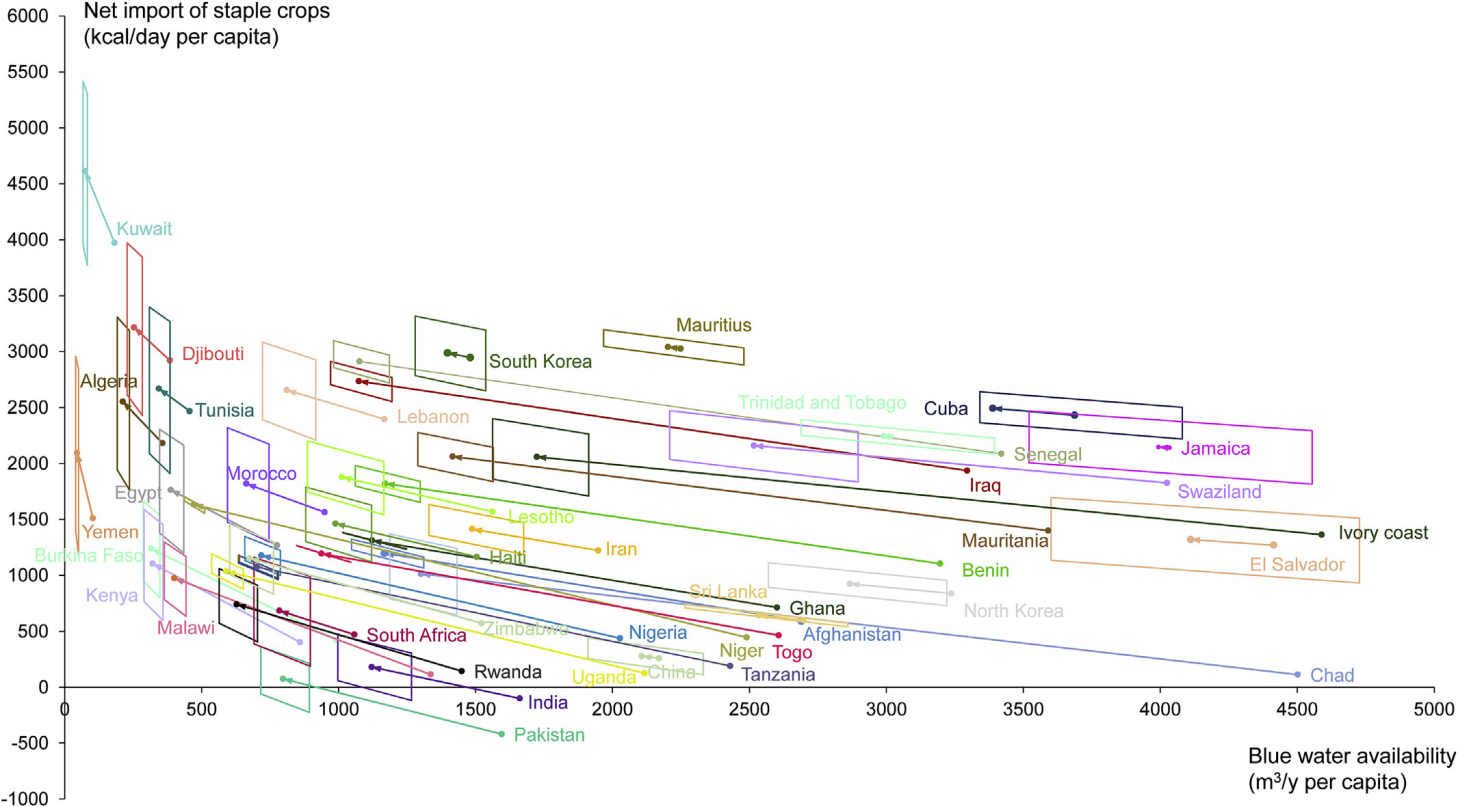
Projection of staple crop imports per country from the 2000s (the lower right dot for each country) to 2050 (the upper left dot). The upper left dot per country is the central projection for 2050 using the medium population scenario and best-fitted curve; each quadrilateral reflects the uncertainty in the central projection as a result of uncertainties in population growth and the shape of the regression curve. The left and right sides of the quadrilateral correspond to the high and low population projection, respectively, and the upper and lower sides of the quadrilateral correspond to the high and low values of the regression slope coefficient (reflecting the 95% reliability interval).

In 2050, when assuming UN's medium population growth scenario, the net import of staple crops in kcal/day per capita is projected to increase for almost all selected countries except for Cuba where net import of staple crops is projected to drop slightly (by 2%). India and Pakistan, the only net exporting counties of staple crops in the list in the period 2001–2010, will become net importers of staple crops by 2050 (Table 1). Water availability per capita will decrease in all countries (Fig. 3). Between the baseline 2001–2010 and the year 2050, the total net import of staple crops for the selected countries in kcal/day is projected to increase by a factor 2.5 for the medium population growth scenario (or a factor 2.2 for the low, or a factor 2.8 for the high population growth scenario) (Supplemental Table 2). In the period 2001–2010, the gross import of staple crops in the selected countries in kcal/year corresponded to 34% of the world total gross export (FAO, 2015). From this, we compute that towards 2050 the overall global trade in staple crops should increase by a factor of 1.4, 1.6 or 1.8, according to the low, medium and high population growth scenario, respectively, in order to meet the increased staple food needs of these most water-scarce countries (Supplemental Table 3). The largest expected relative increases in the net import of staple crops (by a factor of around 30 in the medium population growth scenario) are found for Chad, Malawi and Uganda, that were nearly self-sufficient in the 2000s, but grow fast and are becoming water scarcer rather quickly.

**Table 1:** The average net import of staple crops (2001–2010) in kcal/day per capita, the projected net import of staple crops for the year 2050, and the uncertainties in the projected net import due to uncertainties in population growth and in the shape of the regression curve.

Country	Average net import of staple crops (2001–2010) in kcal/day per capita	Projected net import of staple crops in 2050 with the medium population growth scenario in kcal/day per capita	Uncertainty in projected net import	
			due to uncertainty in population growth (±)	due to uncertainty in the shape of the regression curve (±)
Afghanistan	593	1187	81	13
Algeria	2182	2553	75	697
Benin	1104	1820	73	93
Burkina Faso	421	1238	70	364
Chad	113	997	67	255
China	258	279	72	93
Cuba	2492	2432	73	139
Djibouti	2921	3218	78	693
Egypt	1268	1763	81	473
El Salvador	1269	1320	97	285
Ghana	712	1310	75	2
Haiti	1164	1463	88	249
India	−100	180	86	208
Iran	1222	1414	84	140
Iraq	1937	2736	75	106
Ivory Coast	1363	2060	73	274
Jamaica	2141	2148	93	235
Kenya	403	1103	76	419
Kuwait	3973	4614	75	749
Lebanon	2396	2655	86	353
Lesotho	1570	1879	99	233
Malawi	114	973	71	262
Mauritania	1399	2062	70	151
Mauritius	3026	3041	84	75
Morocco	1564	1819	81	430
Niger	446	1632	57	19
Nigeria	438	1177	65	109
North Korea	837	924	82	111
Pakistan	−419	75	80	215
Rwanda	143	740	80	248
Senegal	2088	2912	68	123
South Africa	471	684	95	394
South Korea	2947	2988	66	269
Sri Lanka	599	643	85	16
Swaziland	1826	2161	98	221
Tanzania	190	1075	72	31
Togo	464	1193	76	1
Trinidad and Tobago	2245	2240	85	71
Tunisia	2468	2668	78	666
Uganda	126	1036	69	88
Yemen	1510	2096	81	805
Zimbabwe	573	1152	84	230

The projected net imports of staple crops in 2050 as shown in Table 1 and Fig. 3 are subject to both uncertainty in population growth and uncertainty in the shape of the regression curve. Uncertainty in population growth directly translates in uncertainty in future water availability per capita; indirectly, this results in uncertainty on staple food import per capita, because food imports depend on water availability per capita following the regression curve. The uncertainties in the projected net import of staple crops per country related to population growth range between 57 and 99 kcal/day per capita, while the uncertainties coming from the curve-shape range from 1 until 805 kcal/day per capita (Table 1). The total uncertainty related to the regression analysis at 95% level of confidence ranges from 219 to 275 kcal/day per capita (Supplemental Table 4). The horizontal extent of each quadrilateral in Fig. 3 reflects the uncertainty in population growth; the vertical extent follows from the upper and lower estimates for the regression curve shape parameters. The shape of the quadrilateral is country dependent. For countries with blue water availability exceeding 1500 m^3^/y per capita in 2050, the quadrilateral is wider horizontally; this means that for those countries the uncertainty in population growth mainly translates in moving horizontally in the graph, and less vertically, because the regression curve has a low slope in this range and uncertainties in the slope are moderate. For countries with less than 1500 m^3^/y of blue water availability per capita, the quadrilateral gets stretched more vertically; this means that the uncertainties in the curve shape become greater than the uncertainties related to population growth. For some countries (China, Cuba, El Salvador, Jamaica, Mauritius, South Korea, Sri Lanka and Trinidad and Tobago), the data point for 2001–2010 is within the surface of the quadrilateral for 2050. This is due to the fact that following the low population scenario, there will be a decrease in the inhabitants of these countries by 2050.

## 4 Discussion

One of the limitations of the study is the focus on blue water availability and exclusion of green water resources. Blue and green water scarcity are naturally related though, so that it is unlikely that countries with low blue water availability per capita are rich in green water resources to produce food. Indeed, in the selected countries staple crops are mainly irrigated, which indicates that rain-fed (green-water-based) agriculture alone is insufficient. Nevertheless, given the relevance of scarcity of green water (Schyns et al., 2015), we recommend future study to further evaluate the potential effect of increasing green water scarcity, or overall green-blue water scarcity, on international food trade.

Another limitation is that total blue water availability per country has been taken as a 30-years average for the period 1961–1990, not accounting for climatic changes where they may have occurred. However, when expressed per capita, the effect of population growth on water availability per capita will be by far dominant in all countries. While precipitation has a high interannual variability, the linear trend for the global average precipitation from the Global Historical Climatology Network during 1901–2005 is statistically insignificant (Bates et al., 2008). Given the strong population growth in all countries considered, both in the past and the future, trends in national water availability per capita will anyhow be dominated by changes in population. However, including climatic changes, particularly for the future, can possibly refine our results.

We found that although a person normally needs 2000–2500 kcal/day, there are countries that are importing over that need from only staple crops already in the period 2001–2010, such as Djibouti, Kuwait, Mauritius and South Korea. Algeria, Lebanon and Tunisia will join these countries by 2050. We may question the validity of our projection method in this range, because once all staple food needs are imported, the precise amounts will probably rather depend on other factors, like dietary preferences and food waste.

Although the regression curve representing the historical relation between net national staple-crop import and national water availability per capita that was used to project net national staple-crop imports in 2050 gets very steep when water availability per capita gets very low, the steepness in the curve is represented by a high number of data points. Two countries are projected for 2050 to fall outside the bounds of the data used to fit the regression, namely Kuwait and Yemen. The results for these two countries should thus be taken with extra caution. The projected net import of staple crops for these two countries together represents less than 3% of the total net import of staple crops of the selected countries, so it does not affect the overall results of the study.

Although war and other socio-political factors have impacted trade of some countries in specific periods (e.g. the economic sanctions for Iraq in the 1990s), there will be no change in the study results if we exclude those countries from the analysis. This has been checked by carrying out the regression analysis without Afghanistan, Iraq and Chad for the relatively recent period 2000–2010. This did not cause significant changes affecting the main conclusions of the paper.

The list of the 42 most water-scarce countries includes some countries that are major exporter of one or more specific types of staple crops. In 2010, China, for instance, was an important exporter of millet, potatoes, rice, sorghum, sweet potatoes and yams; Egypt exports potatoes, rice and sweet potatoes; Ghana and Jamaica export yams; India exports maize, millet, rice and sorghum; Iran exports potatoes; Kenya sorghum; and Mauritius cassava. For rice, exports from the selected water-scarce countries were responsible for 24% of global export in 2010. India, the largest rice exporter, already faces major environmental issues related to the overuse of water resources, particularly groundwater depletion (Gupta and Seth, 2007), which threatens the sustainability of its future production and limits its exporting ability.

Based on an analysis of 42 water-scarce countries over five decades of change we found a significant logarithmic shaped relation between net staple-food import in kcal/day per capita and blue water availability per capita. Most of the selected countries follow the regression curve shape, with an exception for a few anomalously-behaving countries such as India, Pakistan and Sri Lanka. The curve found here has a similar shape as the relation found earlier by Yang et al. (2003), although they considered different countries, less staple crops and a shorter period of change, and looked at kg of import rather than kcal.

## 5 Conclusions

Using the regression curve and UN population growth scenarios, we project that, compared to the average in the baseline period 2001–2010, the total net import of staple crops for the selected countries in kcal/y will increase towards 2050 by a factor of 2.2, 2.5 or 2.8, for the low, medium and high population growth scenario, respectively. This means that global trade in staple foods should increase by a factor of 1.4–1.8 in order to meet the staple food needs of the 42 most water-scarce countries in the world. This finding is of broader interest than for the water-scarce countries only; it indirectly influences all other countries involved in staple crop trade. Amongst others, this raises the question of where additional amounts of staple crops in the future could be sourced from, and what additional water and other environmental impacts that may have in these other countries.

## Contributors

The three authors designed the research, analysed the data and wrote the paper. H.C carried out the calculations.

## Funding

This research did not receive any specific grant from funding agencies in the public, commercial, or not-for-profit sectors.

## Conflict of interest

The authors report no conflict of interest.

## Appendix A Supplementary data

Suppl-Info-Revised Suppl-Info-Revised
