# Supplementary material for: Expected increase in staple crop imports in water-scarce countries in 2050
Source: Water Res X. 2018 Oct 5;1:100001. doi: 10.1016/j.wroa.2018.09.001 (PMC6549899; doi:10.1016/j.wroa.2018.09.001)
Supplement: Suppl-Info-Revised [file mmc1.docx]

Supplementary Information

**Expected increase in staple crop imports in water-scarce countries in 2050**

Hatem Chouchane, Maarten S. Krol, and Arjen Y. Hoekstra

**Supplemental Figure 1.** The fitted regression curve for the relation between the average blue water availability per capita and the net import of staple crops per capita for the selected 42 counties, with average data for the periods 1961-1970, 1971-1980, 1981-1990, 1991-2000, and 2001-2010 (five data points per country).

**Supplemental Figure 2.** The total food supply (kcal/day per capita) of Iraq and Kuwait from 1961 till 2010. Both countries had a drop in their food supply between 1990 and 1991. Kuwait’s food supply has recovered to its levels before the crisis while Iraq is still recovering its food supply after two decades.

**Supplemental Table 1.** Ratios to convert net staple crops import from tonne to kcal. Source FAO (FAO 2001).

| **Crops** | **kcal/tonne** |
| --- | --- |
| Wheat | 3330 |
| Barley | 3332 |
| Rice | 3600 |
| Maize | 3560 |
| Soybeans | 3350 |
| Oats | 3850 |
| Sorghum | 3430 |
| Yams | 900 |
| Cassava | 1090 |
| Potatoes | 700 |
| Sweet potatoes | 970 |
| Rye | 3190 |
| Millet | 3400 |

**Supplemental Table 2.** The average net import of staple crops (2001-2010) in kcal/day and the projected net import in 2050 (both in absolute terms). Increases at aggregate (regional) level in terms of a percentage are shown between brackets.

| Country | Average net import of  staple crops (2001-2010)  in kcal/day | Projected net import of staple crops in 2050 in kcal/day | | |
| --- | --- | --- | --- | --- |
|  |  | Low population growth scenario | Medium population growth scenario | High population growth scenario |
| Algeria | 73403627 | 125317954 | 144134592 | 164213102 |
| Benin | 9190238 | 35487307 | 41039587 | 47010745 |
| Burkina Faso | 5767426 | 45282767 | 52983794 | 61292700 |
| Chad | 1156026 | 29624584 | 35039563 | 40909741 |
| Djibouti | 2287708 | 3321963 | 3817215 | 4339578 |
| Egypt | 95953606 | 225945677 | 266395245 | 310814234 |
| Ghana | 15458876 | 55608046 | 65615419 | 76450294 |
| Ivory coast | 25059086 | 87347681 | 100527984 | 114746186 |
| Kenya | 14469611 | 87756244 | 105381362 | 124711626 |
| Lesotho | 3039597 | 4613072 | 5612744 | 6727715 |
| Malawi | 1484307 | 35696847 | 41981988 | 49427559 |
| Mauritania | 4470855 | 14508538 | 16598265 | 18827522 |
| Mauritius | 3701177 | 3278051 | 3797534 | 4362222 |
| Morocco | 47855747 | 67507239 | 79468789 | 92485975 |
| Niger | 6161593.283 | 104978252.2 | 117866665.7 | 131530680.6 |
| Nigeria | 62132016.37 | 403818145.2 | 469078619.6 | 539171149.7 |
| Rwanda | 1322201 | 12609277 | 15673163 | 19240427 |
| Senegal | 23942934.98 | 93446237.2 | 105484514.8 | 118208428.4 |
| South Africa | 22916689 | 33716727 | 44856677 | 57572318 |
| Swaziland | 2048842 | 3208637 | 3872944 | 4602186 |
| Tanzania | 7574402 | 123802126 | 147359985 | 173074441 |
| Togo | 2633114.175 | 15705597.3 | 18707391.66 | 21989182.12 |
| Tunisia | 25118009 | 31162847 | 35958306 | 41096282 |
| Uganda | 3602919 | 88939542 | 105573480 | 123648228 |
| Zimbabwe | 7533032 | 27950407 | 34107596 | 40932461 |
| **Africa** | **468283640** | **1760633767 (276%)** | **2060933423 (340%)** | **2387384983 (410%)** |
| Afghanistan | 14531396.84 | 55049492.42 | 66442391.89 | 78859579.3 |
| China | 337819482.2 | 252115304.2 | 376238909.7 | 518245613.6 |
| India | -115798546 | 140551272 | 307123147.9 | 504157516.2 |
| Iran | 86180028.2 | 108864039 | 130371566.3 | 154179394.7 |
| Iraq | 53141057.75 | 200053367.6 | 228849673.8 | 259825063.6 |
| Kuwait | 9588944.005 | 24164233.77 | 27332903.39 | 30713937.35 |
| Lebanon | 9394004.242 | 12749338.47 | 14897251.31 | 17241332 |
| North Korea | 19960646.45 | 20140484.37 | 24856555.01 | 30077491.81 |
| Pakistan | -65064622.73 | -1577069.764 | 23330950.75 | 52362703.4 |
| South Korea | 140803145.8 | 134420348.4 | 151181541.2 | 168660678.9 |
| Sri Lanka | 11719972.01 | 10284559.99 | 13398828.77 | 16860989.24 |
| Yemen | 31510181.89 | 84640767.57 | 98886137.01 | 114257244.7 |
| **Asia** | **533785691** | **1041456138 (97%)** | **1462909857 (174%)** | **1945441545 (264%)** |
| Cuba | 28042161.42 | 22046839.52 | 25145748.95 | 28555115.09 |
| Haiti | 10869528.14 | 17200575.15 | 20755913.03 | 24656108.16 |
| Jamaica | 5744292.197 | 4880741.499 | 5820581.881 | 6877726.699 |
| Trinidad and Tobago | 2918633.378 | 2467176.055 | 2892888.017 | 3356511.966 |
| **Caribbean** | **47574615** | **46595332 (-2%)** | **54615132 (15%)** | **63445462 (33%)** |
| El Salvador | 7552439 | 6782198 | 8434237 | 10316708 |
| **Central America** | **7552439** | **6782198 (-10%)** | **8434237 (12%)** | **10316708 (37%)** |
| **Total selected countries** | **1057196385** | **2855467435 (170%)** | **3586892648 (239%)** | **4406588698 (317%)** |

**Supplemental Table 3.** The average gross import of staple crops (in the period 2001-2010) in tonne/y and Gcal/y and the single effect of increased future import of staple crops in the 42 selected water-scarce countries on overall global trade (keeping all other variables equal).

|  | Average import of staple crops (2001-2010) | | Gross import of staple crops in 2050 under increased demand in water-scarce countries alone (Gcal/y) | | |
| --- | --- | --- | --- | --- | --- |
|  | tonnes/y | Gcal/y | Low population growth scenario | Medium population growth scenario | High population growth scenario |
| Selected water-scarce countries | 148218500 | 460500 | 1042200 | 1309200 | 1608400 |
| Rest of the world | 283993200 | 894500 | 894500 | 894500 | 894500 |
| World | 432211700 | 1355000 | 1936770 | 2203740 | 2502929 |
| Share of the selected water-scarce countries in global food import | 34% | 34% | 54% | 59% | 64% |

Source: Trade data for 2001-2010 from FAO (2015). Projected import for selected countries in 2050 based on the totals from Table S2 (multiplied by 365 to convert daily to annual values).

**Supplemental Table 4.** Statistical output (from SPSS) using per country bias (dummy 1-41), the lower and upper bound for B with 95% confidence interval and the total uncertainty in the regression analysis.

| Model | | Unstandardized coefficients | | Standardized coefficients | t | Sig. | 95.0% confidence interval for B | | The total uncertainty in the regression analysis |
| --- | --- | --- | --- | --- | --- | --- | --- | --- | --- |
|  |  | B | Std. Error | Beta |  |  | Lower bound | Upper bound |  |
|  | (Constant) | 6034.082 | 451.240 |  | 13.372 | 0.000 | 5143.212 | 6924.953 |  |
|  | Log (blue water availability) | -712.595 | 65.320 | -0.675 | 10.909 | 0.000 | -841.555 | -583.634 |  |
|  | dummy 1 | 1559.927 | 266.572 | 0.250 | 5.852 | 0.000 | 1033.643 | 2086.212 | 263.1 |
|  | dummy 2 | 1281.239 | 233.790 | -0.205 | -5.480 | 0.000 | -1742.805 | -819.674 | 230.8 |
|  | dummy 3 | 505.087 | 221.514 | 0.081 | 2.280 | 0.024 | 67.759 | 942.415 | 218.7 |
|  | dummy 4 | -646.754 | 228.656 | -0.104 | -2.828 | 0.005 | -1098.184 | -195.324 | 225.7 |
|  | dummy 5 | 608.297 | 245.889 | 0.097 | 2.474 | 0.014 | 122.846 | 1093.748 | 242.7 |
|  | dummy 6 | -3.289 | 234.804 | -0.001 | -0.014 | 0.989 | -466.856 | 460.277 | 231.8 |
|  | dummy 7 | -421.196 | 236.497 | -0.067 | -1.781 | 0.077 | -888.105 | 45.713 | 233.5 |
|  | dummy 8 | -460.281 | 237.489 | -0.074 | -1.938 | 0.054 | -929.148 | 8.586 | 234.4 |
|  | dummy 9 | 646.876 | 234.318 | 0.104 | 2.761 | 0.006 | 184.269 | 1109.483 | 231.3 |
|  | dummy 10 | 450.022 | 276.373 | 0.072 | 1.628 | 0.105 | -95.613 | 995.657 | 272.8 |
|  | dummy 11 | -582.617 | 237.024 | -0.093 | -2.458 | 0.015 | -1050.567 | -114.667 | 234.0 |
|  | dummy 12 | 1726.010 | 264.093 | 0.276 | 6.536 | 0.000 | 1204.619 | 2247.401 | 260.7 |
|  | dummy 13 | -568.261 | 239.810 | -0.091 | -2.370 | 0.019 | -1041.712 | -94.810 | 236.7 |
|  | dummy 14 | 189.861 | 254.969 | 0.030 | 0.745 | 0.458 | -313.517 | 693.239 | 251.7 |
|  | dummy 15 | 1334.301 | 278.953 | 0.214 | 4.783 | 0.000 | 783.572 | 1885.030 | 275.4 |
|  | dummy 16 | -471.321 | 229.285 | -0.075 | -2.056 | 0.041 | -923.992 | -18.650 | 226.3 |
|  | dummy 17 | 234.966 | 254.929 | 0.038 | 0.922 | 0.358 | -268.333 | 738.266 | 251.6 |
|  | dummy 18 | 266.373 | 252.430 | 0.043 | 1.055 | 0.293 | -231.993 | 764.738 | 249.2 |
|  | dummy 19 | 2480.999 | 240.382 | 0.397 | 10.321 | 0.000 | 2006.420 | 2955.578 | 237.3 |
|  | dummy 20 | 72.757 | 253.726 | 0.012 | 0.287 | 0.775 | -428.168 | 573.682 | 250.5 |
|  | dummy 21 | 190.192 | 256.332 | 0.030 | 0.742 | 0.459 | -315.877 | 696.260 | 253.0 |
|  | dummy 22 | -35.990 | 225.849 | -0.006 | -0.159 | 0.874 | -481.878 | 409.898 | 222.9 |
|  | dummy 23 | -72.086 | 221.309 | -0.012 | -0.326 | 0.745 | -509.009 | 364.837 | 218.5 |
|  | dummy 24 | 1578.019 | 263.163 | 0.253 | 5.996 | 0.000 | 1058.464 | 2097.573 | 259.8 |
|  | dummy 25 | -962.458 | 228.573 | -0.154 | -4.211 | 0.000 | -1413.723 | -511.194 | 225.6 |
|  | dummy 26 | 1926.405 | 249.631 | 0.308 | 7.717 | 0.000 | 1433.565 | 2419.244 | 246.4 |
|  | dummy 27 | -445.474 | 236.280 | -0.071 | -1.885 | 0.061 | -911.955 | 21.008 | 233.2 |
|  | dummy 28 | -74.269 | 250.660 | -0.012 | -0.296 | 0.767 | -569.140 | 420.602 | 247.4 |
|  | dummy 29 | 845.093 | 267.057 | 0.135 | 3.164 | 0.002 | 317.849 | 1372.337 | 263.6 |
|  | dummy 30 | 1989.238 | 256.931 | 0.318 | 7.742 | 0.000 | 1481.986 | 2496.490 | 253.6 |
|  | dummy 31 | -22.849 | 245.800 | -0.004 | -0.093 | 0.926 | -508.125 | 462.427 | 242.6 |
|  | dummy 32 | -8.984 | 224.413 | -0.001 | -0.040 | 0.968 | -452.037 | 434.069 | 221.5 |
|  | dummy 33 | 1249.747 | 225.508 | 0.200 | 5.542 | 0.000 | 804.533 | 1694.962 | 222.6 |
|  | dummy 34 | 2222.078 | 246.407 | 0.356 | 9.018 | 0.000 | 1735.603 | 2708.553 | 243.2 |
|  | dummy 35 | 280.385 | 253.479 | 0.045 | 1.106 | 0.270 | -220.052 | 780.822 | 250.2 |
|  | dummy 36 | -264.055 | 240.405 | -0.042 | -1.098 | 0.274 | -738.680 | 210.570 | 237.3 |
|  | dummy 37 | 648.068 | 263.263 | 0.104 | 2.462 | 0.015 | 128.316 | 1167.819 | 259.9 |
|  | dummy 38 | 504.275 | 243.204 | 0.081 | 2.073 | 0.040 | 24.124 | 984.426 | 240.1 |
|  | dummy 39 | 696.749 | 262.011 | 0.112 | 2.659 | 0.009 | 179.467 | 1214.030 | 258.6 |
|  | dummy 40 | 1493.856 | 229.019 | 0.239 | 6.523 | 0.000 | 1041.710 | 1946.002 | 226.1 |
|  | dummy 41 | 1224.604 | 230.877 | 0.196 | 5.304 | 0.000 | 768.791 | 1680.418 | 227.9 |
|  | | | | |  |  |  |  |  |

**Supplementary references**

FAO (2001) Food balance sheets: A handbook, Food and Agriculture Organization of the United Nations (FAO), Rome, Italy.

FAO (2015) FAOSTAT Online Database. Statistics Division, Food and Agriculture Organization of the United Nations (FAO), Rome, Italy.
